# A full-length *S1* gene sequencing of a novel emerged GI-19 and GI-23 lineages of *Infectious bronchitis virus* currently circulating in chicken flocks in upper Egypt reveals marked genetic diversity and recombination events

**DOI:** 10.1186/s12985-025-02718-6

**Published:** 2025-05-07

**Authors:** Eman Shosha, Sara Abdelnaser, Ali Mahmoud Zanaty

**Affiliations:** 1 Virology department, Faculty of Veterinary Medicine, New Valley University, El-Kharga, Egypt; 2 Gene Analysis Unit, Reference Laboratory for Quality Control on Poultry, Agriculture Research Center (ARC), Animal Health Institute, Giza, Egypt

**Keywords:** *Infectious bronchitis virus*, Variants, Spike gene, Sequencing, Broilers, Layers, QX strain

## Abstract

**Background:**

*Infectious bronchitis virus* (IBV) is a highly contagious evolving pathogen that causes respiratory, urinary and reproductive symptoms; threatening the poultry industry globally.

**Methods:**

During this study, 90 tissue specimens were collected from various poultry flocks of seven Upper Egypt governorates from 2023 to 2024 for genetic characterization.

**Result:**

Typical IBV lesions of the inoculated embryos in the specific-pathogen-free-embryonated chicken eggs (SPF-ECE) were observed. Using real-time reverse transcriptase polymerase chain reaction (rRT-PCR) assay targeting the conserved N gene, only 60 samples were considered positive with 66.6%. Collectively, 23 tissue specimens were examined through a one-step PCR assay. Sequencing is targeting the *S1* gene, and the phylogenetic analysis was conducted based on partial sequencing showed that *Avian coronavirus* (ACoV) isolates belong to GI-23 (n = 18), GI-12 (n = 2), GI-1 (n = 1), and GI-19 (n = 2). Genotyping of the *S1* gene indicates that GI-23 shows a genetic similarity to Egyptian isolates, and Israeli variants with nucleotide identity percentages (95–97%) and, (88–92%); respectively. Concerning full sequencing, five ACoV isolates were clustered as GI-23 (n = 3), and GI-19 (n = 2). Currently, QX-strains showed low genomic relatedness with Egyptian strains, and vaccinal strains with nucleotide (78–79%), and amino acid (77–80%), and (75–77%) identities, respectively. This is the first detailed study that recorded various IBV isolates, especially the novel emerged QX strain circulating in chicken flocks in Egypt. The recombination event within the Assuit-1-QX-EGYIBV-2024 isolate was detected as a result of recombination between the major (GI-19) and minor (GI-22) putative parents. Importantly, the G1-19 and G1-23 shared multiple amino acids mutations at *S1* gene.

**Conclusions:**

This study provides empirical evidence for the ACoV circulating in Egypt in vaccinated and non-vaccinated poultry flocks despite the excessive vaccination schemes.

## Introduction

Avian infectious bronchitis (IB) is a respiratory viral infection of an economically major concern for poultry health worldwide, causing significant economic losses by negatively affecting meat and egg production [1]. Infectious bronchitis disease is caused by *Infectious bronchitis virus* (IBV) which is designated as *Avian coronavirus*-ACoV, belongs to genus *Gammacoronavirus,* species Avian coronavirus, of the *Orthocoronavirinae* subfamily, *Coronaviridae* family (https://ictv.global/taxonomy; accessed on 12 January 2024) and it affects a variety of organs including respiratory, reproductive, nervous, digestive, and urinary systems of chickens as well as it affects egg productivity, hatchability, and shell quality in laying hens [2–4]. ACoV is an emerging, highly contagious virus that infects all ages of chickens with a morbidity rate of 100% and mortality rate can reach 80% in accordance to the birds' age, their immune status, the circulating IBV strains and following secondary infections or mixed infections with other pathogens [2, 5, 6].

*Avian coronavirus* is an enveloped, unsegmented, and positive single-stranded RNA virus with a length of approximately 27.6 kb. in size which encodes four major structural proteins consisting of the spike (S) glycoprotein, envelope (E), membrane (M), nucleocapsid (N) proteins, besides accessory proteins which are not essential for viral replication [3a, 3b, 5a, 4b, 4c, 5a, 5b, and putative 7 protein (6b)] [7–10]. The ACoV spike protein cleaved into subunits S1 and S2 with approximately 535 and 625 amino acids, respectively. The S1 glycoprotein, contains three hypervariable regions (HVRs 1, 2, and 3), forming the extracellular part of the virus and it is associated with viral attachment, virus neutralization, tissue tropism, and serotype-specific epitopes, and is a principal target of neutralizing antibodies [11–13]. During ACoV replication and evolution, the mutation rate continuously occurs in the genome especially in hypervariable regions of the S1 glycoprotein resulting in significant genotypic, pathogenic, and antigenic variations worldwide; which enable the emergence of newly variant strains and triggering ACoV outbreaks [8, 14]. Subsequently, based on the nucleotide sequencing of the currently available *S1* gene, the genotyping of ACoV has mostly been conducted [15, 16]. Accordingly, prevalent nucleotide diversity including continuous genetic mutation and recombination was shown in this region containing three rapidly evolved HVRs (amino acids 38–67, 91–141, and 274–387) [17]. Based on genetic studies, more than 30 ACoV serotypes have been identified worldwide with limited cross-protection to each other [18, 19]. Regarding *S1* gene sequencing analysis, ACoV strains are recently clustered into seven major genotypes (GI–GVII), with 35 distinct lineages, and several inter-lineage recombinants. Moreover, the genotype GI possesses the majority of genetic lineages, representing Mass and 793B lineages [20–22]. In addition, the ACoV lineage GI-23 (also named Variant 2) has spread and emerged with severe respiratory infections in various countries including the Middle East, Africa, Asia and Europe [23–25].

In Egypt, recent reports record the emergence of various ACoV genotypes in different localities, necessitating ongoing surveillance and specific genetic characterization in local poultry flocks [26–33]. Notably, ACoV: GI-23, including field ACoV variants, became the most important lineage circulating in Egypt. The GI-23 was first reported in Egypt in 2001, also known as Egyptian Variant-I, and it was genetically similar to the Israeli variant strain IS/720/99 [34]. Subsequently, a new distinct variant strain within GI-23 emerged and called Egyptian variant-II [35]. Taken into account, other ACoV lineages were co-circulated in Egypt such as GI-12 [36], GI-13 [37] and GI-16 (identified as Q1 like strains) [38]. Importantly, the ACoV -QX strain, clustered in the GI-19 lineage, became the predominant field strain in several countries as Pakistan, China, Korea, Kurdistan-Iraq, and also Europe [39, 40]. Additionally, the QX ACoV genotype was antigenically different from both classical vaccinal and other variant strains [41]. The QX strain is mostly recognized for its nephropathogenic effects, proventriculitis, and impaired egg productivity; causing outbreaks in affected flocks globally with high mortality rates [42, 43].

Despite mass vaccination strategies in Egypt, the continuous evolution of ACoV variants in different localities remains a major economic concern for the local poultry industry. Taken together, the current study aims to genetically characterize recent field ACoV strains circulating in Upper Egypt governorates and determine the genetic divergence comparable to currently available vaccines. Surprisingly, we present the first report of full *S1* gene sequencing of ACoV -QX field strains (GI-19 lineages) as well as variant strains belonging to GI- 23 lineage; providing monitoring data about genetic diversity, molecular and evolutionary characteristics of these circulating ACoV strains in the respective Egyptian farms.

## Materials and methods

### Ethical statement

This study protocol adhered closely to the ethics and guidelines for Animal Experimentation set in accordance with the New Valley Research Ethics Committee of the faculty of veterinary medicine, New Valley University under the reference number (04-2023-200249).

### History of examined flocks and clinical samples

In the period between 2023 and 2024, a total number of 90 samples from 90 various poultry flocks (broilers, breeders and layers) suffered from the clinical forms of the disease as respiratory manifestations such as (sneezing, coughing, bronchial rales, and gasping), egg production disorders, and elevated mortality and morbidity rates, were originated from seven Upper Egypt governorates (Beni-Seuf, El-Mina, Assuit, Sohag, Qena, Aswan, New Valley). These diseased examined flocks exhibited necropsy pictures as tracheitis, congested lung, congested trachea with caseous exudates, turbidity of air sacs, pale enlarged kidneys, and urolithiasis. In addition, degenerated ovaries and swollen oviducts associated with egg peritonitis were noticed in layers. Mostly, some investigated flocks have previously been vaccinated with various ACoV vaccines. The descriptive epidemiological data of the examined flocks is summarized for ACoV detection in (Table 1). The tissue specimens were collected as pooled homogenate from the trachea, kidney, lung, and oviduct each flock then stored at −80° C till used for virus isolation and polymerase chain reaction (PCR) test. Moreover, the collected tissue samples were prepared as 10% suspension in sterile phosphate-buffered saline (PBS) (pH 7.4) including 10% antibiotics solution (penicillin1000 IU/mL, streptomycin 1 mg/mL, gentamycin 2 mg/mL, Sigma Chemical Co., USA) then the homogenates were cooled centrifuged at 3000 rpm for ten minutes after incubation at 4 °C overnight; the supernatants were finally gathered for further IBV analysis. The differences among groups in farm’s number, age, breed, bird’s number, and collected samples were performed using non-parametric Kruskal–Wallis test after checking for normality of the distribution using Shapiro–Wilk normality test. The *P*-value (≤ 0.05) was considered to be statistically significant.

**Table 1:** The descriptive epidemiological data of the examined flocks for ACoV detection in the seven Upper Egypt governorates

Flock location	Poultry farms	Flock type	Flock age	Year	Vaccination programs	Mortality %	No. of birds	Collected samples				Signs and postmortem lesions
Beni-Seuf	10	Broilers,	20–35 days	2023	H120 (GI-1) + 4/91(GI-13) + Inactivated vaccine (GI-1)	9	9000–12000	Lung	Trachea	Kidney	Oviduct	Severe respiratory manifestations, congested lung, and trachea with caseous exudates
		Breeders	23–61 weeks				3000–7000	44	31	15	–	
El-Mina	18	Broilers,	23–31 days	2023	IB Primer (GI-1,12) + 4/91(GI-13) + Inactivated vaccine (GI-1)	10	7000–10,000	43	32	12	10	Respiratory manifestations, egg production disorders, nephropathogenic lesions, congested lung, and trachea, degenerated ovaries, and swollen oviducts
		Breeders Layers	22–60 weeks				4000–9000					
Assuit	29	Broilers	25–35 days	2023 2024	IB Primer (GI-1,12) + IB VarII (GI-23) + Inactivated vaccine (GI-1)	10	12,000–15000	60	23	17	35	
		Breeders, layers	22–62 weeks				9000–15000					
Sohag	12	Broilers	20–30 days	2023 2024	H120 (GI-1) + IB VarII (GI23) + Inactivated vaccine (GI-1)	8	9000–11000	50	23	7	13	
		Breeders layers	23–62 weeks				2000–7000					
Qena	7	Broilers	23–35 days	2024	H120 (GI-1) + 4/91 (GI-13) + Inactivated vaccine (GI-1)	9	8000–12000	29	20	9	5	
		Breeders Layers	22–61 weeks				4000–8000					
Aswan	6	Broilers	22–33 days	2023	IB Primer (GI-1,12) + 4/91(GI-13) and some are non-vaccinated	8	5000–7000	10	12	8	–	Severe respiratory manifestations, congested lung, and trachea with caseous exudates
New Valley	8	Broilers	20–35 days	2024	IB Primer (GI-1,12) + IB VarII (GI-23) and some are non-vaccinated	8	4000–6000	36	25	15	–	

### Virus isolation assays

Tissue homogenate (0.2 mL) of each pooled sample was inoculated into 9–11-day-old SPF-ECE originated from Nile SPF (Koom Oshiem, Fayoum, Egypt) via the allantoic cavity as described by [44], then incubated at 37 °C for seven days with continuous daily candling. Then, we checked the dead eggs for the evidence of the embryonic changes as stunting, curling, dwarfing, and urates deposition. The un-inoculated SPF-ECE was kept as a negative control. Consequently, the allantoic fluids were harvested for further subsequent passages. Also, up to three serial passages were performed for each pooled specimen.

### RNA extraction and real-time PCR analysis

The genomic viral RNA was extracted directly from the harvested allantoic fluid (n = 90) using QIAamp Viral RNA Mini Kit (Qiagen, Hilden, Germany) following the manufacturer’s recommendations. Further, ACoV identification was conducted by rRT-PCR focusing on the most conserved N gene targeting 130 base pair (pb) fragment using QuantiTect® probe RT-PCR kit (Qiagen, Hilden, Germany), with specific oligonucleotide primers [45].

### S1 gene sequencing and phylogenetic analysis

Specifically, the *S1* gene of twenty three positive isolates was partially amplified and 5 out of 23 ACoV-positive isolates were selected for full *S1* gene amplification and sequencing using a Qiagen one-step RT-PCR Kit (Qiagen, Hilden, Germany) according to the manufacturer’s instructions through specific oligonucleotide primer pair sets (Table 2). The various PCR amplicons of different sizes were visualized by electrophoresis on a 1.5% agarose gel electrophoresis and, after that, purified using the QIAquick Gel Extraction kit (Qiagen, Hilden, Germany) following the manufacturer’s protocol. Notably, to further characterize the genetic evolution of newly detected ACoV strains, the purified PCR products were subsequently sent to the Macrogen Clinical Laboratory (South Korea) for bidirectional sequencing to perform partial and full-length *S1* gene sequencing in both directions. Furthermore, the obtained nucleotide sequences of the twenty-three positive ACoV isolates were submitted to GenBank with their accession numbers (Table 3). The oligonucleotide and amino acid sequences were compared and aligned with other related strains of published ACoV vaccines and reference strains existed representing all ACoV genotypes in the GenBank database using the Clustal W program [46]. Finally, the phylogenetic tree was constructed using the maximum likelihood method employing the Kimura 2-parameter model via MEGA software version 7.0 and BioEdit software packages, with levels evaluated using 1000 bootstrap replicates [47].

**Table 2:** Consensus primer sets applied for the amplification of the Egyptian ACoV isolates

Sequencing type of the whole S1 gene	ACoV -forward Primer sequence (5’–3’)	ACoV- reverse Primer sequence (5’–3’)	Product size in (bp)	References
ACoV-HVR1-3	GTK TAC TAC TAC CAR AGT GC	CAG AYT GCT TRC AAC CAC C	79–928	[48]
Full-length	AGTBTCYACACAGTGTTAYAAGCG	GGYCTRWANKSRCTYTGGTAG	1591 bp	[49]
	TTAAATCATTTCAGTGTGTTAATAAT	CATAACTAACATAAGGGCAA	1704 bp

**Table 3:** Recent ACoV isolates data used for partial and full-length spike glycoprotein *S1* gene sequencing analysis

No	Isolate identification	Accession No	Genotype	Flock type and location
1	**Assuit-1-QX-EGYIBV-2024**^**a**^	**PQ093635**	**GI-19**	**Layers, and broilers, Assuit governorate**
2	**Assuit-2-QX-EGYIBV-2024**^**a**^	**PQ093636**	**GI-19**	**Layers, and broilers, Assuit governorate**
3	**NewValley-1-EGYIBV-GI23-2023**^**b**^	**PQ093637**	**GI-23**	**Broilers, New Valley governorate**
4	**Minya-2-EGYIBV-GI23-2024**^**b**^	**PQ093638**	**GI-23**	**Breeders, El-Mina governorate**
5	**BeniSeuf-3-EGYIBV-GI23-2024**^**b**^	**PQ093639**	**GI-23**	**Broilers, BeniSeuf governorate**
6	Sohag-EGYIBV-1-GI1-2023	PQ093640	GI-1	Breeders, Sohag governorate
7	Assuit-1-EGYIBV-GI23-2023	PQ093641	GI-23	Breeders, Assuit governorate
8	Assuit-2-EGYIBV-GI23-2023	PQ093642	GI-23	Broilers, Assuit governorate
9	Assuit-3-EGYIBV-GI23-2023	PQ093643	GI-23	Breeders, Assuit governorate
10	Sohag-4-EGYIBV-GI23-2023	PQ093644	GI-23	Broilers, Sohag governorate
11	Sohag-5-EGYIBV-GI23-2023	PQ093645	GI-23	Broilers, Sohag governorate
12	NewValley-6-EGYIBV-GI23-2023	PQ093646	GI-23	Broilers, New Valley governorate
13	NewValley-7-EGYIBV-GI23-2023	PQ093647	GI-23	Broilers, New Valley governorate
14	Mina-8-EGYIBV-GI23-2023	PQ093648	GI-23	Breeders, El-Mina governorate
15	Mina-9-EGYIBV-GI23-2023	PQ093649	GI-23	Broilers, El-Mina governorate
16	Beniseuf-10-EGYIBV-GI23-2023	PQ093650	GI-23	Broilers, Beni-seuf governorate
17	Beniseuf-11-EGYIBV-GI23-2023	PQ093651	GI-23	Breeders, Beni-seuf governorate
18	NewValley-12-EGYIBV-GI23-2024	PQ093652	GI-23	Broilers, New Valley governorate
19	Sohag-13-EGYIBV-GI23-2024	PQ093653	GI-23	Breeders, Sohag governorate
20	Aswan-14-EGYIBV-GI23-2024	PQ093654	GI-23	Broilers, Aswan governorate
21	Aswan-15-EGYIBV-GI23-2024	PQ093655	GI-23	Broilers, Aswan governorate
22	Assuit-1-EGYIBV-GI12-D274-2023	PQ093656	GI-12	Breeders, Assuit governorate
23	Sohag-/GI-12/D274-35/2023	PQ093657	GI-12	Broilers, Sohag governorate

Our twenty-three Egyptian isolates are presented as follows: Bold isolates 1–2 are clustered as GI-19, bold isolates 3–5 are clustered as GI-23, Isolate 6 is clustered as GI-1 Isolates, 7–21 are clustered as GI-23, and finally isolates 22–23 are clustered as GI-12. Subsequently, a: *S1* gene of IBV-GI-19 isolates (full sequencing). b: *S1* gene of IBV-GI-23 isolates (full sequencing)

### Antigenicity prediction and homology modelling

To further identify the three HVRs and the mutations in the newly representative ACoV isolates, the three-dimensional structure (3D) of GI-19 isolates and GI-23 S1 proteins were designed by homology-modelling approach. Moreover, the automatic mode of Swiss Institute of Bioinformatics (SWISS-MODEL) web server (SWISS-MODEL, https:// swissmodel.expasy.org/ accessed on 01 December 2023) was employed to investigate the homologous sequences of the target proteins and generate an experimentally 3D structures [50].). Subsequently, proteins showing the high amino acid similarity and extensive coverage were selected as templates for homology modeling. The obtained model was identified and visualized with Chimaera software [51], plotting the evolutionary features of the protein. These modeled protein structures of illustrative isolates from each lineage were recognized and visualized using PyMOL software (Version 1.7.4, LLC) (http://www.pymol.org/).

### ACoV evolutionary analysis

The *S1* gene sequences of the current isolates were accomplished and iteratively screened in barrels with various available ACoV genotypes such as GI-1, GI-19, GI-12, and GI-23 to check any new recombinant ACoV strains. Regarding recombination events, the RDP4 analysis (version 4.97) was accomplished [52] using different methods with their default parameters to indicate potential recombination events. Particularly, the recombination events analysis was considered proven as a preliminary scan if identified by a minimum of 7 algorithms (RDP, GENECONV, BootScan, MaxChi, Chimaera, SiScan, and 3Seq) as well as the calculated* p*-value was less than 1.0 × 10–30 [53].

## Results

### Clinical findings and mortality incidence

**Fig. 1:**
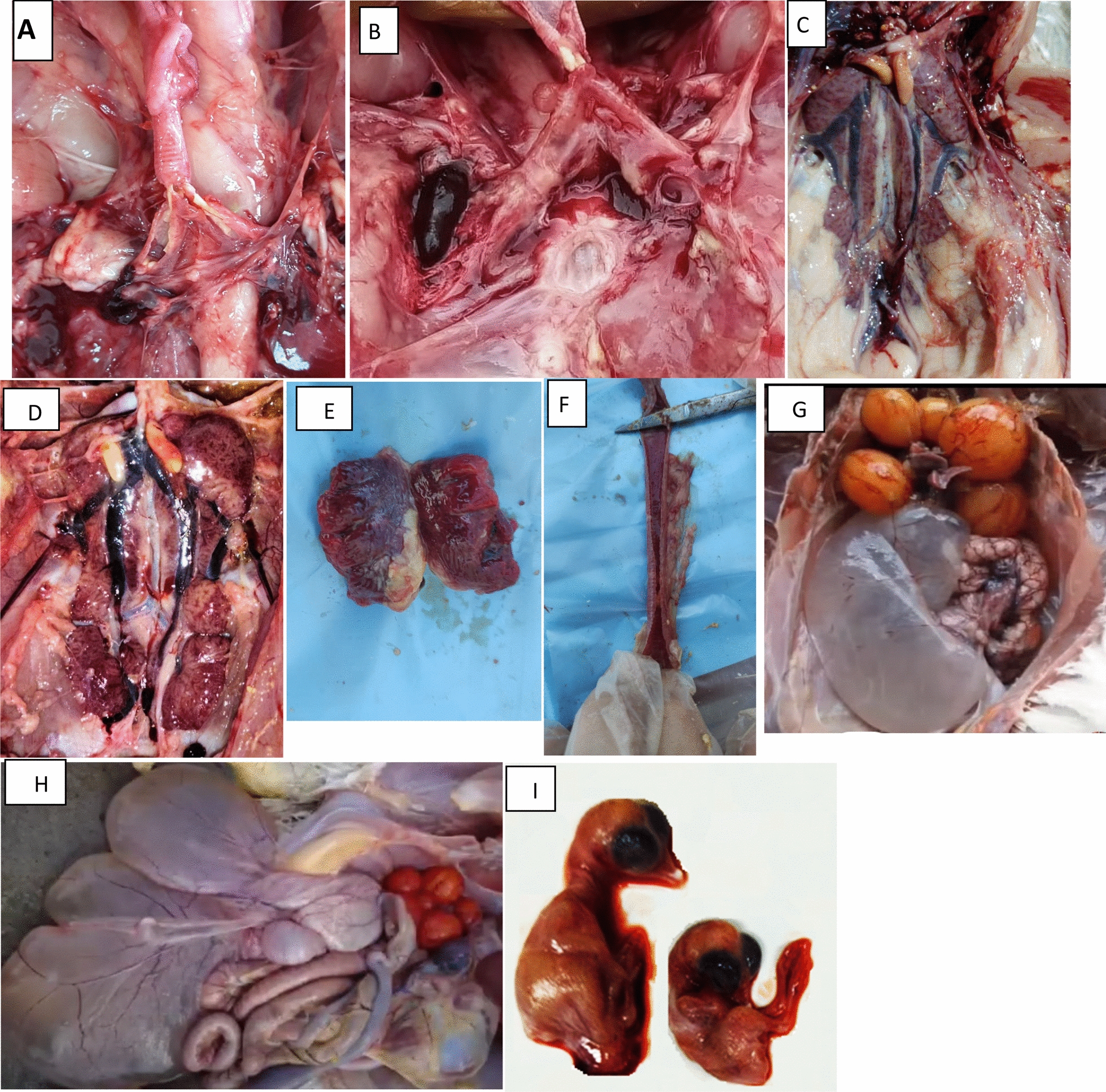
Macroscopic lesions from internal organs of broiler and layer chicken infected with variant ACoV strains.** A, B, F** Broiler chicken naturally infected with ACoV; showing severe hemorrhagic trachea containing caseous plugs. **C, D** kidneys were congested and swollen with urate deposits. **E** Congested lung. **G, H** Layer chicken naturally infected with ACoV -GI-19; showing dilatation and suffusion of the oviduct, and deformation of the ovarian follicles. **I** Normal embryo on the left side and curled, stunted, and dwarfed embryos infected with ACoV on the right side

Regarding positive poultry flocks, ACoV was detected in 60 farms out of 90 with a prevalence rate of 66.6%. Clinical symptoms in the various examined flocks in the seven Upper Egypt governorates, were respiratory manifestations, egg production disorders, and high mortality and morbidity rates. Notably, the macroscopic findings were mainly tracheitis, congested lung, congested trachea with caseous exudates, pale enlarged kidneys, as well as degenerated ovaries and swollen oviducts observed in layers (Fig. 1). The mortality rate was reported at 10% whereas the morbidity rate was approximately 80%. Specifically, our observed findings recorded that ACoV was isolated from both vaccinated and non-vaccinated flocks as 18 isolates (78.2%) were from vaccinated flocks with classic and variant vaccines while the other 5 isolates (21.7%) belonged to non-vaccinated ones. There was no any significant values observed among groups.

### Gross findings

Necropsy of dead birds mostly revealed hemorrhagic tracheitis, a caseous plug in the trachea, lung congestion, air sacs cloudiness, nephritis, swollen enlarged congested kidneys, and urolithiasis (Fig. 1). Moreover, the ovaries showed abnormal oviduct and ovarian follicle development.

### ACoV isolation on SPF-ECE and molecular identification

**Fig. 2:**
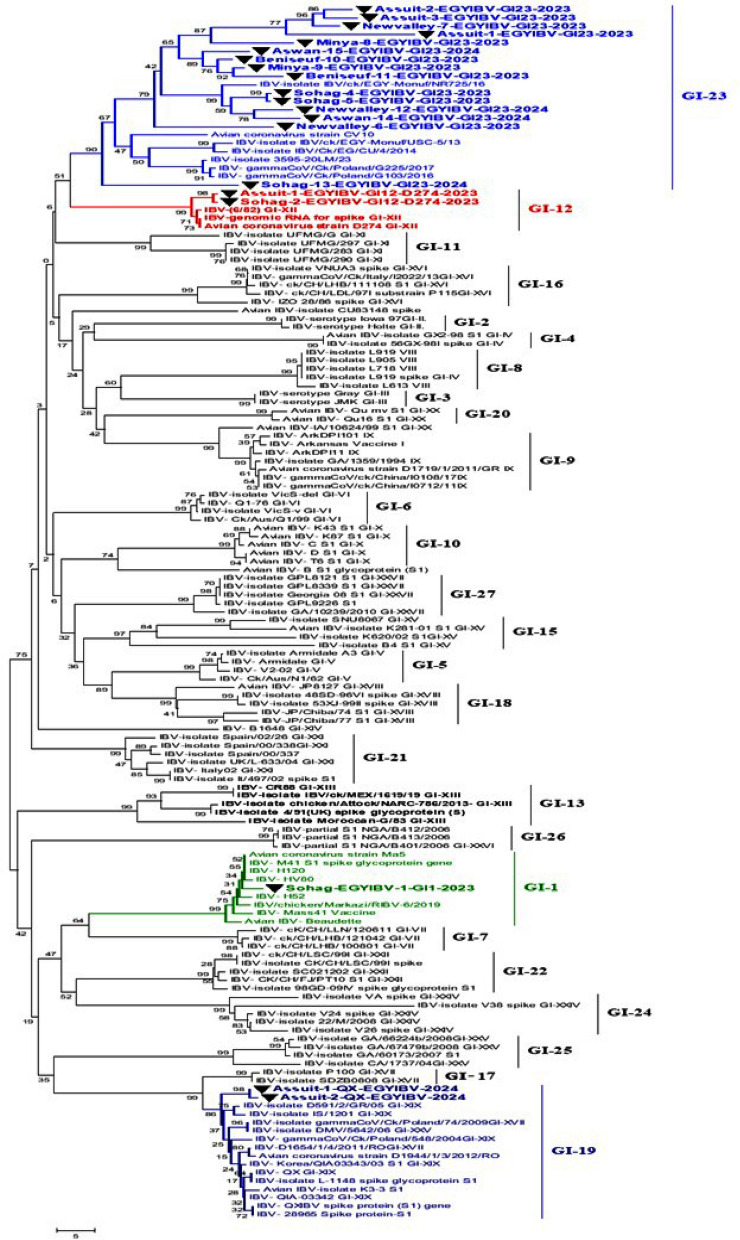
Partial sequencing phylogenetic tree were constructed using the nucleotide sequence alignments of *S1* gene of various ACoV strains with other reference strains and Egyptian strains. The phylogenetic analysis of *S1* gene revealing that 25 ACoV isolates (indicated by black triangle) located in various genotypes as follow: (fifteen isolates labelled with blue color clustered as GI-23, two isolates labelled with red color clustered as GI-12, one isolate labeled with green colour clustered as GI-1 and the last two isolates labelled with blue color clustered as GI-19). The tree was constructed by the Maximum likelihood method with 1,000 bootstrap replicates, using MEGA 7.0 software

After three to five successful passages of ACoV in the SPF-ECE, typical ACoV lesions of the inoculated embryos were observed; including embryo curling, stunting, dwarfing, subcutaneous hemorrhage, ureates deposition, hemorrhagic spots on the liver and swollen kidneys (Fig. 1). On the other hand, using rRT-PCR assay targeting the conserved N gene, only 60 samples out of 90 were considered positive with a 66.6% (Table 1). Furthermore, only 23 positive samples were amplified with a one-step RT-PCR assay for *S1* gene partial sequencing and molecular characterization (Fig. 2 and Table 3).

### Sequencing and phylogenetic analysis of the ACoV-S1 gene

**Fig. 3:**
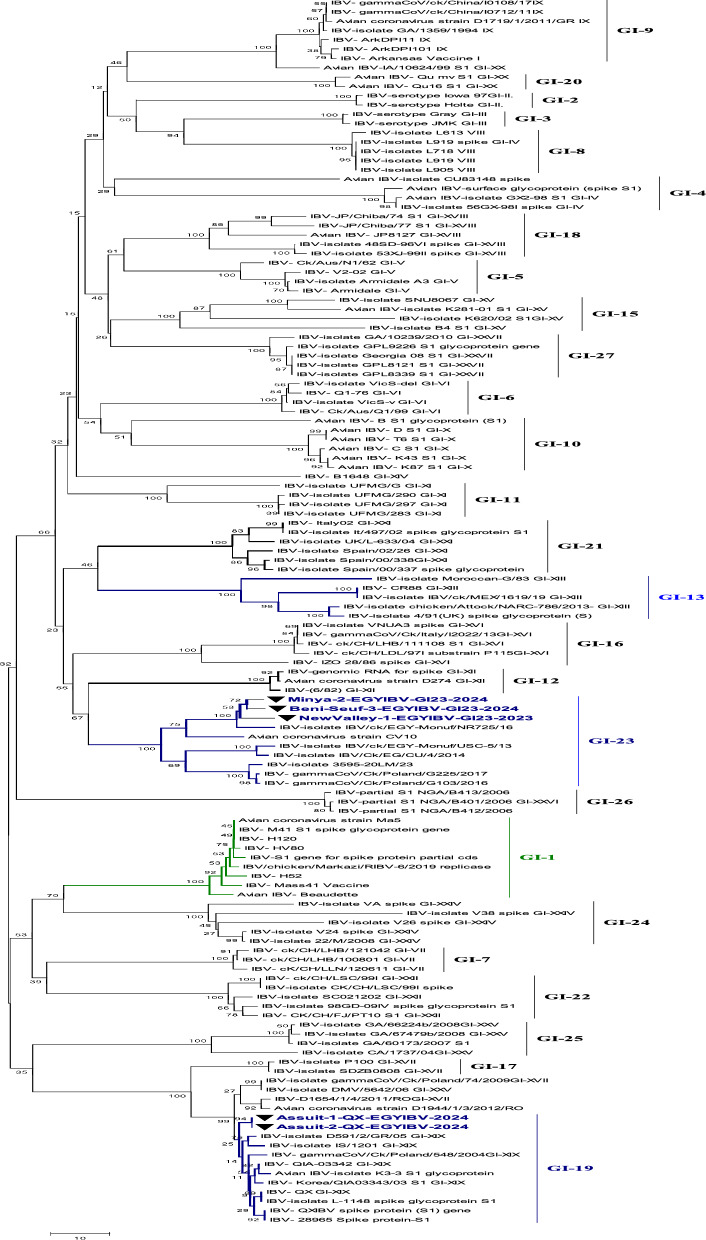
A ccollective phylogenetic tree was constructed using the nucleotide sequence alignments of Full-length *S1* gene sequencing (1610 Nucleotide) in comparison to other reference strains including Egyptian strains. The phylogenetic analysis of the *S1* gene revealed that 5 ACoV isolates (labelled with blue colour and black triangle) were located in various genotypes as follows: (three isolates clustered as GI-23, two isolates clustered as GI-19). The tree was constructed by the Maximum likelihood method with 1,000 bootstrap replicates, using MEGA 7.0 software

**Fig. 4:**
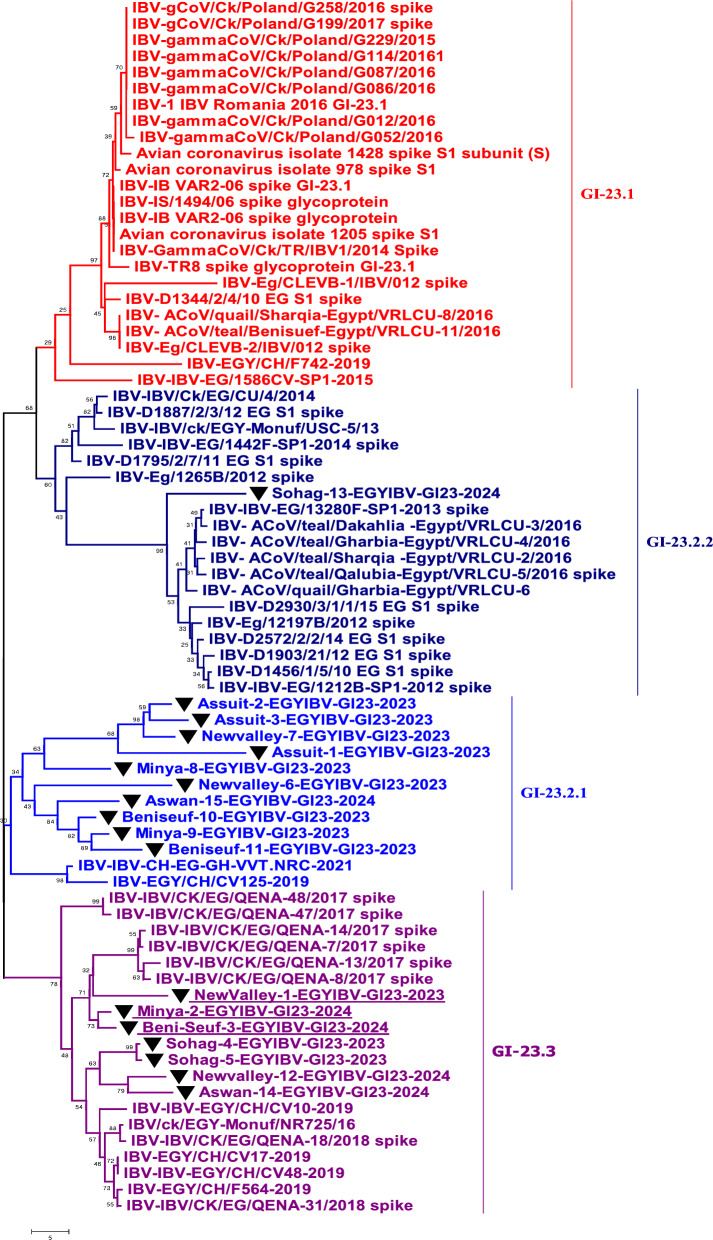
Detailed phylogenetic tree of ACoV strains within genotype GI-23 based on full *S1* gene sequences sequencing comparable to other referential strains deposited in Genbank. The phylogenetic analysis of the *S1* gene resulted in establishing three sub-clades of 18 ACoV isolates (indicated as black triangle) located in the GI-23 genotype as follows: (ten isolates clustered as GI-23.2.1, one isolate clustered as GI-23.2.2, and other seven isolates clustered as GI-23.3). The tree was constructed by the Maximum likelihood method with 1,000 bootstrap replicates, using MEGA 7.0 software

Collectively, 23 ACoV isolates were examined through PCR assay. A PCR-amplified product at 880 bp targeting the HVRs-1-3 of the *S1* gene was detected from all 23 positive ACoV isolates for partial sequencing (Fig. 2). Besides, five PCR-positive amplicons out of 23 specimens were also investigated for full *S1* gene sequencing (Fig. 3). Phylogenetic analysis was conducted among various ACoV strains deposited in Genbank based on the analysis of the partial and full-length *S1* gene to differentiate between the field and vaccinal strains as well as for accurate genotyping (Tables 4, 5). Importantly, the construction of the phylogenetic tree based on partial sequencing showed that eighteen Egyptian ACoV isolates belong to genotype I clade 23 (GI-23), two isolates clustered as GI-12, one isolate clustered as GI-1, and the last two novel isolates classified as GI-19 (Fig. 2). Within the GI-23, the fifteen isolates were submitted to the Genbank database under accession numbers (PQ093641- PQ093655). Interestingly, our isolates including Sohag-13-EGYIBV-GI23-2024, Aswan-14-EGYIBV-GI23-2024, and Aswan-15-EGYIBV-GI23-2024 have the highest genetically related to IBV-EG/1212B-SP1-2012, IBV/ck/EGY-Monuf/NR725/16, and IBV/Ck/EG/CU/4/2014 (Egyptian isolates), with nucleotide identity percentage 97%, 95%, 95%, and on the amino acid level were with 95%, 92%, 91%; respectively (Fig. 2). Furthermore, Sohag-EGYIBV-1-GI1-2023 of accession number PQ093640 were closely genetically similar to IBV- H120, and IBV- Mass41 Vaccine (vaccinal strains), with nucleotide identity percentages of 99%, 97%; respectively, and on the amino acid level was 98%, 95%; respectively. Likewise, Assuit-1-EGYIBV-GI12-D274-2023 and Sohag-2-EGYIBV-GI12-D274-2023 (PQ093656- PQ093657) have the greatest genetically correlated to IBV-D274-GI-XII**,** with nucleotide identity percentage 99% as well as on the amino acid level were with 77% (Table 4). Meanwhile, Sohag-13-EGYIBV-GI23-2024, Aswan-14-EGYIBV-GI23-2024, and Aswan-15-EGYIBV-GI23-2024 isolates were distinctly apparent from IBV- H120, IBV- Mass41, IBV-isolate 4/91(UK.), and IBV-QX, GI-XIX; shared (77–79%), (76–78%), (76–78%), and (75–77%) similarity. Similarly, Assuit-1-EGYIBV-GI12-D274-2023, Sohag-2-EGYIBV-GI12-D274-2023, and Sohag-EGYIBV-1-GI1-2023 also have low homology with IBV-isolate 4/91, and IBV-QX, GI-XIX with nucleotide identity percentage (76–77%) as well as on the amino acid level was with 77% (Table 4). Taken into account, amino acid sequencing analysis of the *S1* gene of Sohag-13-EGYIBV-GI23-2024, Aswan-14-EGYIBV-GI23-2024, and Aswan-15-EGYIBV-GI23-2024 isolates revealed 88% similarity to each other, also 92% identity based on nucleotide identity level. Whereas, Assuit-1-EGYIBV-GI12-D274-2023 and Sohag-2-EGYIBV-GI12-D274-2023 showed 100% homology to each other, based on amino acid, and nucleotide identity levels. Meanwhile, Sohag-13-EGYIBV-GI23-2024, Aswan-14-EGYIBV-GI23-2024, and Aswan-15-EGYIBV-GI23-2024 isolates were distinctly apparent from IBV- H120, IBV- Mass41, IBV-isolate 4/91(UK.), and IBV- QX, GI-XIX; shared (77–79%), (76–78%), (76–78%), and (75–77%) similarity. On the other hand, the phylogenetic trees design based on full sequencing (Fig. 3) of five Egyptian ACoV isolates were clustered as GI-23 (three isolates of accession number PQ093637- PQ093639), and GI-19 (two isolates of accession number PQ093635- PQ093636). Notably, NewValley-1-EGYIBV-GI23-2023, Minya-2-EGYIBV-GI23-2024, and Beni-Seuf-3-EGYIBV-GI23-2024 isolates shared (97–99%), (95–97%) nucleotide similarity with IBV-isolate IBV/ck/EGY-Monuf/NR725/16, and IBV/CK/EG/QENA-47/2017 as well as on the amino acid level were with (96–97%), (95–96%); respectively (Table 5). Moreover, Assuit-1-QX-EGYIBV-2024 and Assuit-2-QX-EGYIBV-2024 have the highest genetically related to IBV- QX, GI-XIX with nucleotide, and amino acid identity percentages of 97%. Meanwhile, NewValley-1-EGYIBV-GI23-2023, Minya-2-EGYIBV-GI23-2024, Beni-Seuf-3-EGYIBV-GI23-2024 isolates were distinctly apparent from IBV- QX, GI-XIX; shared 76%, 77%, 77% similarity. Additionally, Assuit-1-QX-EGYIBV-2024 and Assuit-2-QX-EGYIBV-2024 showed low homology with IBV- Eg/CLEVB-1/IBV/012, IBV- Mass41 vaccine, and IBV-EG/1212B-SP1-2012, with nucleotide identity percentage of 78%, as well as on the amino acid level was with (78–80%), (77–79%), (75–77%), 75%; respectively (Table 5). Also, NewValley-1-EGYIBV-GI23-2023, Minya-2-EGYIBV-GI23-2024, Beni-Seuf-3-EGYIBV-GI23-2024 isolates showed 99% homology to each other, based on amino acid, and nucleotide identity levels. Besides, amino acid and nucleotide sequencing analysis of Assuit-1-QX-EGYIBV-2024 and Assuit-2-QX-EGYIBV-2024 isolates revealed 100% similarity to each other. In particular, the GI-23 eighteen isolates (Fig. 4) were clustered as follows: Sohag-13-EGYIBV-GI23-2024 in subclade GI-23.2.2, Assuit-1-EGYIBV-GI23-2024, Assuit-2-EGYIBV-GI23-2024, Assuit-3-EGYIBV-GI23-2024, Newvalley-6-EGYIBV-GI23-2024, Newvalley-7-EGYIBV-GI23-2024, Minya-8-EGYIBV-GI23-2024, Minya-9-EGYIBV-GI23-2024, Benisuef-10-EGYIBV-GI23-2024, Benisuef-11-EGYIBV-GI23-2024, Aswan-15-EGYIBV-GI23-2024 in subclade GI-23.2.1, Newvalley-1-EGYIBV-GI23-2023, Newvalley-12-EGYIBV-GI23-2024, Benisuef-3-EGYIBV-GI23-2024, Minya-2-EGYIBV-GI23-2024, Sohag-4-EGYIBV-GI23-2023, Sohag-5-EGYIBV-GI23-2023, Aswan-14-EGYIBV-GI23-2024 in subclade GI-23.

**Table 4:**
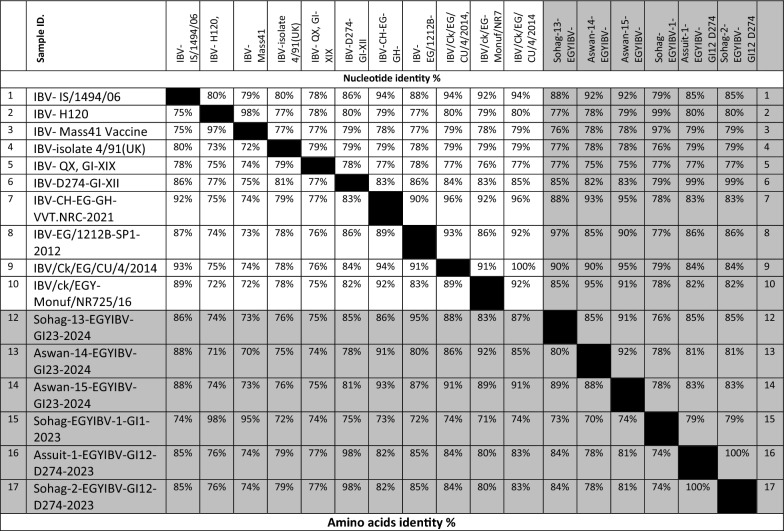
Nucleotide identities and divergence of partially sequenced ACoV isolates comparable to other selected Egyptian and referential strains

Amino acids and Nucleotide identities and divergence of our partially sequenced ACoV isolates comparable to other selected strains including vaccinal strains. The table utilizes a comparative alignment of the *S1* gene in which, the *S1* nucleotide identity percentage of our six Egyptian isolates (GI-23, GI-1, GI-12) ranges from 79 to 100% comparable to other referential strains. Besides, the amino acids identity percentage of these isolates ranges from 97 to 100% comparable to various referential strains

**Table 5:**
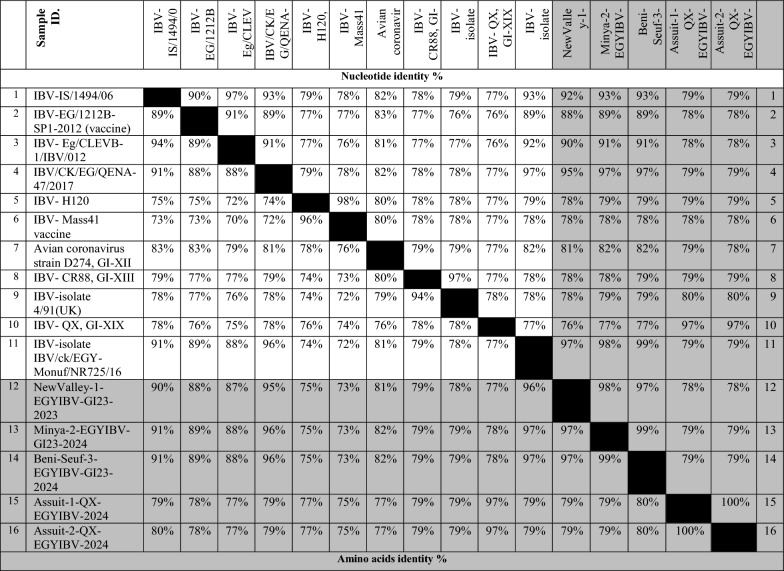
Nucleotide and amino acids identities of full-length sequenced ACoV isolates comparable to other selected Egyptian and referential strains

Nucleotide and amino acids identities of our full-length sequenced ACoV isolates comparable to other selected strains. The table includes a comparative alignment of the *S1* gene in which, the *S1* nucleotide and amino acids identity percentages of our five Egyptian isolates (GI-23, GI-19) range from 99 to 100% comparable to other referential strains

### Analysis of the recombination events

**Fig. 5:**
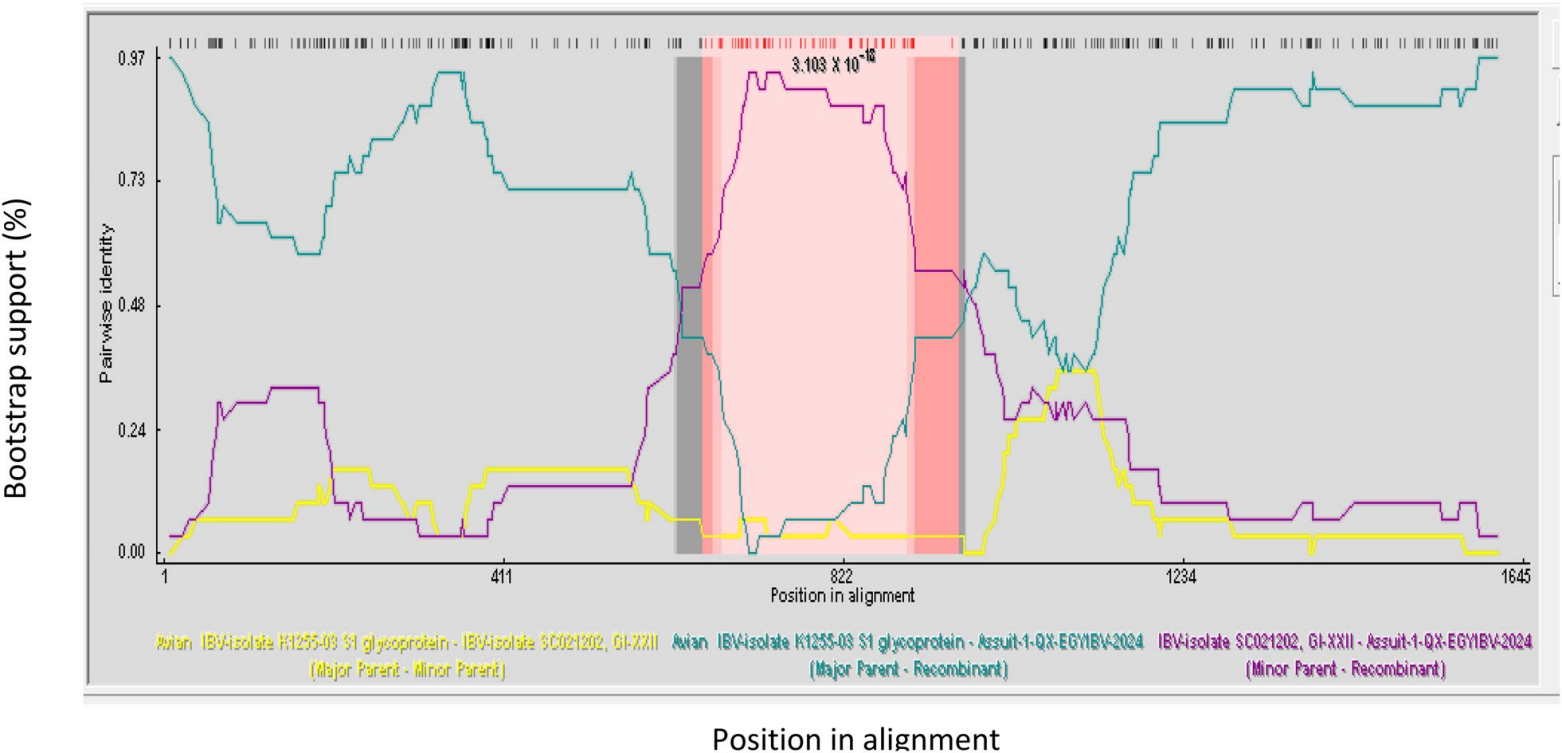
Genetic recombination analysis of the Egyptian IBVAssuit-1-QX-EGYIBV-2024 strain built in the RDP4 software. Pale pink is a track of sequence with a recombination origin. Potential putative parents involved in the recombination events of strain IBVAssuit-1-QX-EGYIBV-2024 are color-coded as follows: The K1255-03 Korean isolate (GI-19), “variant strain" of accession number: AY790364 is a major parent with green colour. SC021202 GI XXII strain, Chinese isolate G1-22 (of GenBank accession no. EU714029) is a minor parent with violet colour. Additionally, Recombinant strain Assuit-1-QX-EGYIBV-2024 with yellow colour

Numerous ACoV variants have been categorized and the genetic recombination events occurred frequently between field viruses predicted inside the same genotype (intra-genotypic), among diverse genotypes (inter-genotypic), and used vaccines. We further detected the possibility of recombination events within our Egyptian ACoV isolates and other genotypes built in the RDP4 software analysis. The results demonstrated a recombinant event resulting in the emergence of a new recombinant strain in the current study. The genetic recombinant IBV Assuit-1-QX-EGYIBV-2024 isolate could be a result of recombination between two strains, the major putative parent belongs to K1255-03 Korean isolate (GI-19) variant strain of an accession number AY790364, whereas the minor putative parent is closely related to the GI-22 lineage, SC021202 Chinese isolate of GenBank accession no. EU714029 (Fig. 5). Different breakpoints were detected in the *S1* gene and the novel recombinant virus (Assuit-1-QX-EGYIBV-2024) may be a precursor for further detectable variants. Specifically, further studies are essential to determine the pathobiological and clinical features of the probably emerged virus in the chicken sector. Besides, continuous sequencing for the currently circulating ACoV strains is substantially recommended to investigate the possible virus spreading, and highlight the diversification tendency along the spike protein regions for a good understanding of its phylogenetic relatedness to other available viruses.

### Homology modeling analysis of S1 protein

**Fig. 6:**
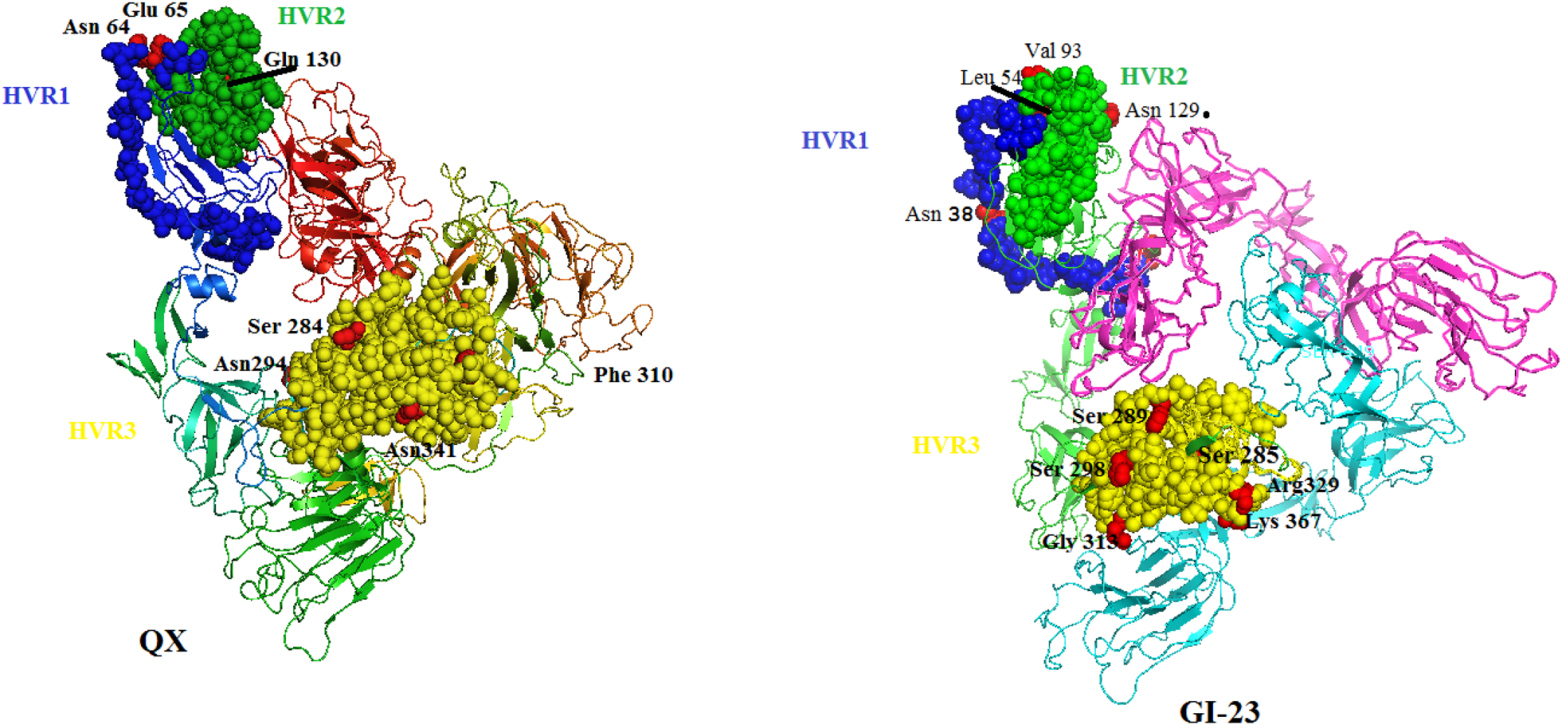
Comparative analysis and characterization of the predicted tertiary structure revealed the specific mutations in the S1protein of the novel identified IBV strains (GI-23 and GI-19). Briefly, a three-dimensional structural analysis structure template was downloaded from the PDB database, and the HVRs regions of IBVs GI-23 and GI-19 isolates compared to referential and vaccinal strains; were predicted. The deep red color indicates the localization of specific mutations in the *S1* protein of GI-23 and GI-19 isolates. The 3D structure was visualized by PyMOL software

Compared with the 3D structure of reported ACoV isolates in the PDB database (PDB ID: 6CV0), prominent obvious amino acid substitutions were located in the major receptor-binding domain (Fig. 6) and adjacent to three HVRs regions. Specifically, the analysis of the S1 protein modelling indicated that the Assuit-1-QX-EGYIBV-2024 strain shared several unique amino acids mutations (aas), such as Asn 64, and Glu 65 in HVR-1, Glu 130 in HVR-2, the Asn 294, Asn 341 Ser 284 and Phe 310 in HVR-3, compared to their parental origin (SC021202 GI XXII Chinese isolate of G1-22) (Fig. 6). In addition, the G1-23 showed substitutions at residues Asn 38 in HVR-1, Len 54, Val 93, Asn 129 in HVR-2, Ser 298, Ser 289, Ser 285, Arg 329, Gly 313, Lys 367 HVR-3 with the representative parental K1255-03 Korean strain. Surprisingly, the current mutations of these residues mainly were leading to the changes in the structure of the S1 protein; causing a change in the ACoV antigenicity and also facilitating the emergence of the variant strains. Interestingly, the results revealed that the Assuit-1-QX-EGYIBV-2024 strain originated from GI-19 and GI-22, however, phylogenetic tree conduction clustered it in GI- 19 and the main S protease cleavage site (HRRRR) was similar to that in GI-19.

## Discussion

Poultry’s’ health is affected by various viral infections, which can be fatal. Over the last 100 years, most of the viral outbreaks have been caused by RNA viruses. Among the RNA viruses, ACoV possess the highest mutation rates [4, 54, 55]. ACoV is one of the major viral pathogens and a significant threat to the poultry industry worldwide. ACoV is enzootic extremely causing enormous economic losses in Egypt as there were many circulating ACoV serotypes and genotypes have been reported. Despite intensive routine vaccination with various live and inactivated vaccines, the ACoV serious outbreaks still occurred across different districts of Egypt. Increasing reports of outbreaks in the poultry populations especially vaccinated ones, highlighting the suboptimal protection offered by vaccines [30, 33]. Particularly, ACoV strains can undergo high genetic variations such as recombination and mutations; contributing to the emergence of novel genotypes and/or serotypes that might be antigenically different from the existing vaccine strains [56, 57]. Furthermore, ACoV genotyping is based on *S1* gene sequencing, especially in the three HVRs regions. These HVRs regions of the *S1* gene encode serotype-specific determinants and harbour the antigenic epitopes essential for the induction of protection [8, 58]. Interestingly, this study targets the phylogeographic characterization and evolutionary relationships of all available ACoV isolate sequences in Upper Egypt.

The current data provides intensive interesting findings of ACoV strains screening in various poultry flocks; recording and confirming novel IBV-QX field strains (GI-19 lineages) in Egypt. As depicted in our results, the positive flocks with a prevalence rate of 66.6% in seven Upper Egypt governorates have generally shown clinical remarkable manifestations and post-mortem findings with a mortality rate of 10%. These current results were in line with previously reported clinical manifestations by [31, 32, 57, 59–61]. Concerning SPF-ECE inoculation, ACoV characteristic pathognomonic lesions were recorded in the inoculated embryos after 3–5 consecutive passages to produce a reliable cytopathic effect. These findings came in accordance with various previous studies [59, 62, 63]. More importantly, rRT-PCR is the most appropriate method for the molecular detection of new emerging ACoV variants; supplying epidemiological data and evolutionary relationship of ACoV strains [18, 59]. Notably, the rRT-PCR results revealed that only 60 samples were considered positive with a percentage of 66.6%. These present molecular results are nearly similar to those [31, 60, 64] who reported the prevalence of positive PCR specimens were 69%, 57% and 64%; respectively]**.**

Specifically, to characterize and further prove the ongoing genetic evolution of the *S1* gene in our ACoV recent isolates, they were partially and fully genome sequenced, and then compared with other reference sequences. The phylogenetic analysis based on partial sequencing revealed that ACoV isolates (n = 23) clustered as three distinct genotypes. Also, the key difference between these current lineages was highlighted through the determination of cleavage recognition motifs of the* S1* gene [65, 66]. In recent years, the GI-23 lineage has been the major ACoV genotype circulating in poultry flocks, in Egypt [28, 48, 49]. In the same line, genotyping was accomplished by phylogenetic analysis of the *S1* gene indicating that GI-23 shows a strong genetic similarity to Egyptian isolates, with nucleotide identity percentages 95–97%. In contrast, Sohag-EGYIBV-1-GI1-2023 was closely genetically similar to vaccinal strains, with nucleotide identity percentages of 97–99%, and the amino acid level was 95–98%. Also, Sohag-13-EGYIBV-GI23-2024, Aswan-14-EGYIBV-GI23-2024, and Aswan-15-EGYIBV-GI23-2024 shared nucleotide identity percentages of 88–92% with IS/1494/06 (Israeli variant) and based on the amino acid identity, the percentages were (86–88%); respectively. Our findings are consistent with [29] who reported that these ACoV isolates (variant-II) were closely related to Eg/12120S/2012 (Egyptian isolate), IS/885/00 and IS/1494/06 (Israeli variants). Similarly, previous studies have also demonstrated the prevalence of variant ACoVs which is closely related to Israeli variants and original Egyptian variants (Egypt/Beni-Suif/01) [27, 28, 35, 67]. In addition, the similarity of our Egyptian ACoV isolates with others from neighbouring countries like Israel may be attributed to the uncontrolled movement of inhabitants and smuggling across border regions.

Lineage of GI-23 was first documented in Israel in 1998 then spread rapidly to Egypt, Middle East countries, and European countries [36, 68, 69]. Collectively, the detailed tree was designed to distinguish the different subclades within genotype GI-23 including subclades GI-23.1, GI-23.2.1, GI-23.2.2, GI-23.3. GI-23.2.2 harbours other viruses in Egypt during 2010–2016. While, GI-23.1 contains strains of EGY-Var1 and IS-Var2 in different countries including Israel, Egypt, Turkey, and Poland between 2010 and 2017. [30]. Regarding our available results, the GI-23 eighteen isolates were clustered as GI-23.2.1 (n = 13), GI-23.2.2 (n = 1), and GI-23.3 (n = 7). Our subsequent results came in accordance with [30] who indicated that all five ACoV isolates belong to GI-23 involving subclades GI-23.2.1, GI-23.2.2, and GI-23.1. Comparable to the commonly available vaccines in Egypt, GI-23 (n = 3) were distinctly apparent from (GI-1)[IBV-H120, IBV-Mass41], and (GI-13) [IBV-4/91] shared different levels identities (75–79%). Our previous observations also align with those findings documented by [31] who mentioned that the nine isolates displayed broad variety levels of nucleotide (77.4–82.3%) and amino acid (74.2–81.8%) similarities to the ACoV vaccines. Besides, the vaccine strain D274 (GI-12) had a far distant relation to 4/91 with nucleotide and amino acid identities (76–77%). The high sequence variations between our isolates and the commercial vaccine strains in Egypt, exhibiting the reason for the vaccine's failure against emerging challenges with the circulating field strains. Thus, the improvement of broadly cross-reactive vaccines against currently emerging ACoV genotypes is urgently warranted.

Regarding our promising results, this is the first detailed study that recorded various ACoV isolates, especially the novel emerged QX strain in all Upper Egypt sectors. The phylogenetic trees based on the complete *S1* gene sequencing demonstrate a novel IBV-QX-Egyptian strains (GI-19) as it is a major genotype in Asia, and Europe, causing serious economic losses to the poultry industry [70, 71]. Assuit-1-QX-EGYIBV-2024 and Assuit-2-QX-EGYIBV-2024 showed low genomic relatedness with IBV-IS/1494/06, IBV- Eg/CLEVB-1/IBV/012, IBV-EG/1212B-SP1-2012 (Egyptian strains), and Mass41, H120, (vaccinal strains), with nucleotide identity percentage of 78–79%, as well as on the amino acid level was with (77–80%), and (75–77%). These subsequent results are not in agreement with [72] who revealed that the AH-2020-QX-Chinese strain shared 86.61% nucleotide identity with the commercial vaccine strains H120 and 4/91. On the contrary, these phylogeny findings agree practically with previous studies conducted by [63, 65] who reported that his ACoV isolates belong to the GI-19, similar to isolates from Thailand and Saudi Arabia. In addition, [73] recorded that JP/Nagasaki/2013, JP/Kochi/2013, and JP/Nagasaki/ 2016 strains belonged to distinct genotypes of GI-13 and GI-19 depending on the full S1 gene sequencing.

Subsequently, the results demonstrated a recombinant event within the novel-identified ACoV Assuit-1-QX-EGYIBV-2024 isolate as a result of recombination between the major and the minor putative parents. This potential recombination indicates various changes occurring in the S1 subunit that might alter the neutralizing epitopes; resulting in the existence of novel ACoV serotypes or genesis of highly virulent strains. Our results came nearly in accordance with [37] who reported the recombination events in IBV/CH/SA/7/2019 as it is emerging due to recombination between GI-23 and GI-13 lineages. Additionally, [49] confirmed the multiple recombination breakpoints in Egyptian field strain CU/4/2014, especially at the 3′ one-third of the genome. Parallel to our results, [36, 73–75] stated that most of the potential recombination breakpoints of field ACoV strains happen in the intermediate region among HVR1, HVR2, and HVR3.

Importantly, the Assuit-1-QX-EGYIBV-2024 strain and the G1-23 shared multiple amino acid mutations in the three HVRs compared to their parental origin. These findings are nearly in agreement with [37] who recorded that IBV/CH/SA/1/2019, IBV/CH/SA/2/2019, and IBV/CH/SA/3/2019 isolates showed substitutions at multiple residues comparable to their parental 4/91 genotype. Concerning previous studies, the changes in a few aa residues on S protein subsequently affect the pathogenicity of the entire ACoV strains [72, 76]. The residues of our G1-19 and G1-23 at positions 38, 54, 64, and 65 are critical for binding the ACoV spike protein to the respiratory tract receptors. Our findings align with [8, 76] who indicated that amino acids 19–272 of the M41 spike are adequate for binding to trachea epithelium and alpha-2,3-linked sialic acids. Similarly, the residues of our G1:23 at position 93 in HVR 2 are critical to establish kidney tissue binding, this observation is also stated by [76].

## Conclusions

Our promising results elucidated that the GI-19-type ACoV has evolved in Upper Egypt governorates as novel emerging QX strains, as well as variant strains belonging to GI-23 lineage. The QX-strains showed low homology with Egyptian strains, and vaccinal strains with nucleotide identity (78–79%). Whereas, GI-23 displays a genetic similarity to Egyptian, and Israeli isolates with nucleotide identity percentages (95–97%) and, (88–92%); respectively. The phylogenetic analysis of *S1* gene indicating that recent ACoV strains showed a comparable evolution rate with other ACoV isolates. The study provides empirical evidence for the ACoV circulating in Egypt in vaccinated and non-vaccinated poultry flocks despite the excessive vaccination schemes. Furthermore, the generated data can be utilized for the adoption of more effective control measures and assistance with improved techniques for efficient vaccine matching and vaccine strain selection for preventing and controlling ACoV. Notably, it is critical to complete the genetic characterization especially full genome sequencing of recent circulating ACoV isolates to study the genetic relatedness among viruses and vaccine strains. Likewise, further studies on the circulating QX strain are needed to provide broad information on their genetic evolution, emergence, and molecular and evolutionary characteristics as there are no commercial vaccination strains currently being applied in this lineage.

## Data Availability

Not applicable.
