# Correction: A full-length S1 gene sequencing of a novel emerged GI-19 and GI-23 lineages of Infectious bronchitis virus currently circulating in chicken flocks in upper Egypt reveals marked genetic diversity and recombination events

**DOI:** 10.1186/s12985-025-02806-7

**Published:** 2025-06-11

**Authors:** Eman Shosha, Sara Abdelnaser, Ali Mahmoud Zanaty

**Affiliations:** 1 Virology department, Faculty of Veterinary Medicine, New Valley University, El-Kharga, Egypt; 2 Gene Analysis Unit, Reference Laboratory for Quality Control On Poultry, Agriculture Research Center (ARC), Animal Health Institute, Giza, Egypt

**Correction** **:** **Virol J 22, 135 (2025)**

**https://doi.org/10.1186/s12985-025-02718-6**

In this article [1], Tables 4 and 5 appeared incorrectly and have now been corrected in the original publication. For completeness and transparency, the old incorrect versions are displayed below.

**Incorrect** Tables 4 and 5

**Table 4:** Nucleotide identities and divergence of partially sequenced ACoV isolates comparable to other selected Egyptian and referential strains

e	h	n	m	de
w	r	g	f	f

Amino acids and Nucleotide identities and divergence of our partially sequenced ACoV isolates comparable to other selected strains including vaccinal strains. The table utilizes a comparative alignment of the S1 gene in which, the S1 nucleotide identity percentage of our six Egyptian isolates (GI-23, GI-1, GI-12) ranges from 79 to 100% comparable to other referential strains. Besides, the amino acids identity percentage of these isolates ranges from 97 to 100% comparable to various referential strains

**Table 5:** Nucleotide and amino acids identities of full-length sequenced ACoV isolates comparable to other selected Egyptian and referential strains

f	e	n
g	g	n

Nucleotide and amino acids identities of our full-length sequenced ACoV isolates comparable to other selected strains. The table includes a comparative alignment of the S1gene in which, the S1 nucleotide and amino acids identity percentages of our five Egyptian isolates (GI-23, GI-19) range from 99 to 100% comparable to other referential strains

**Correct** Tables 4 and 5

**Table 4:**
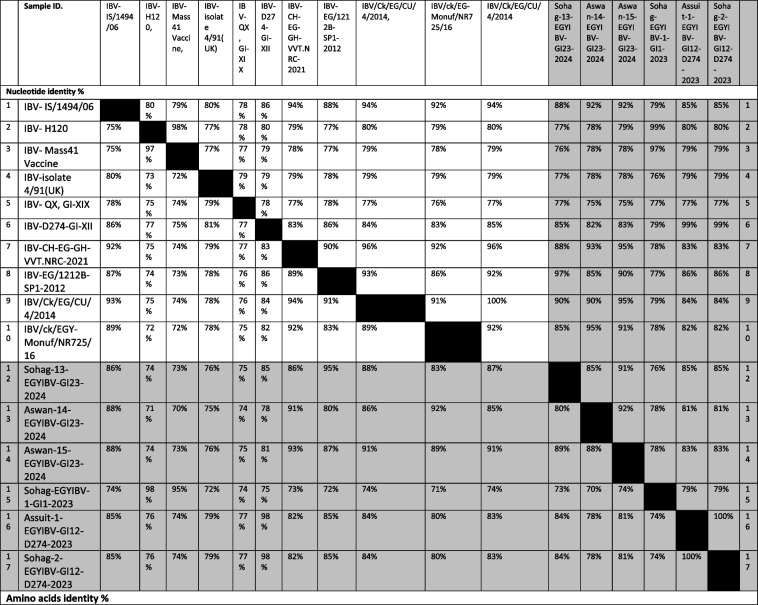
Nucleotide identities and divergence of partially sequenced ACoV isolates comparable to other selected Egyptian and referential strains

Amino acids and Nucleotide identities and divergence of our partially sequenced ACoV isolates comparable to other selected strains including vaccinal strains. The table utilizes a comparative alignment of the S1 gene in which, the S1 nucleotide identity percentage of our six Egyptian isolates (GI-23, GI-1, GI-12) ranges from 79 to 100% comparable to other referential strains. Besides, the amino acids identity percentage of these isolates ranges from 97 to 100% comparable to various referential strains

**Table 5:**
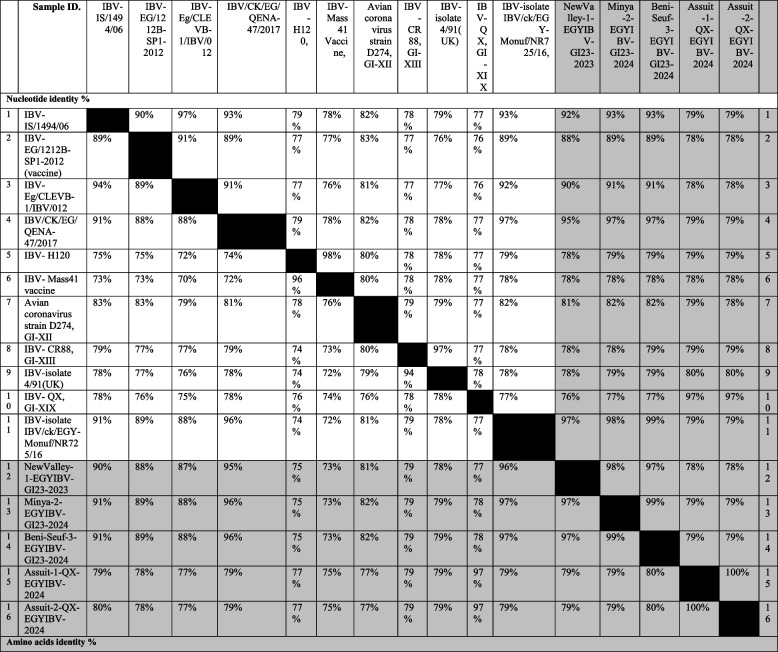
Nucleotide and amino acids identities of full-length sequenced ACoV isolates comparable to other selected Egyptian and referential strains

Nucleotide and amino acids identities of our full-length sequenced ACoV isolates comparable to other selected strains. The table includes a comparative alignment of the S1gene in which, the S1 nucleotide and amino acids identity percentages of our five Egyptian isolates (GI-23, GI-19) range from 99 to 100% comparable to other referential strains

The original article has been corrected.
